# Supplementary material for: Identification of Freezing-Responsive microRNAs and Their Targets in Chinese Jujube by Small RNA and Degradome Sequencing
Source: Int J Mol Sci. 2026 Sep 4;27(17):7912. doi: 10.3390/ijms27177912 (PMC13566890; doi:10.3390/ijms27177912)
Supplement: Supplementary file 1 [file ijms-27-07912-s001.zip › Supplementary Material S2/novel_miR_187.pdf]

Provisional ID	:	novel_miR_187
Score total	:	2259.8
Score for star read(s)	:	3.9
Score for read counts	:	2254.1
Score for mfe	:	0.2
Score for randfold	:	1.6
Score for cons. seed	:	
Total read count	:	4433
Mature read count	:	2549
Loop read count	:	0
Star read count	:	1884

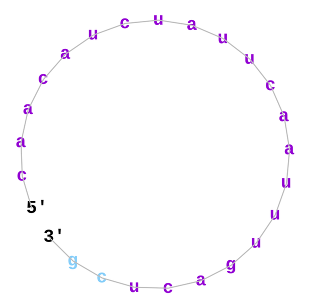

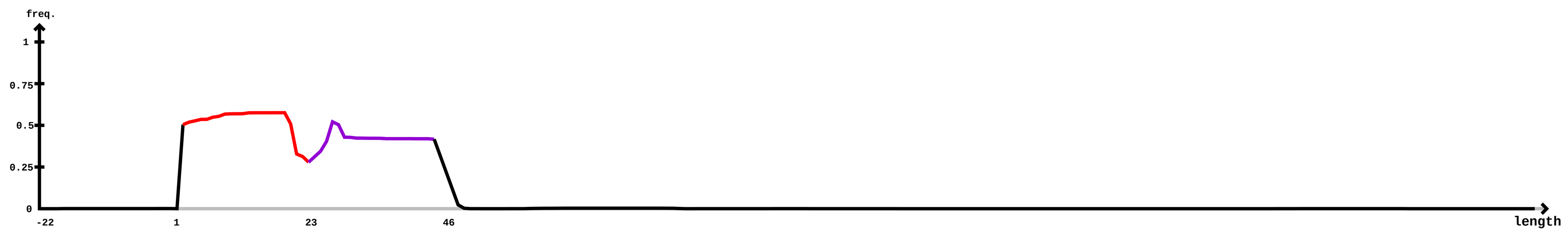

Mature Star

```
5'- aauuuuugaagauguauugagcagucaaauugaauggaugauugcaacaucuauucaauuugacucguuuuuuaucaucaaauuggcauacucacugaaacuuuuuggugacaaauaggaccacacaggacuagaaaucacccauuacugaagaagcaucaggacugaaaacagaagaagacagcgagacaaauggaggcccacacagaagacuuaaaacagaagagaccaauaaaaaggaccaaaaaag -3'  obs
    aauuuuugaagauguauugagcagucaaauugaauggaugauugcaacaucuauucaauuugacucguuuuuuaucaucaaauuggcauacucacugaaacuuuuuggugacaaauaggaccacacaggacuagaaaucacccauuacugaagaagcaucaggacugaaaacagaagaagacagcgagacaaauggaggcccacacagaagacuuaaaacagaagagaccaauaaaaaggaccaaaaaag       exp
    ....(((((.((((....(((((((((((((((((((((.......)))))))))))))))))).)))).)))).)))))((((....(((.(((...((((((((((....(((..(......)..)))....))))).....((((.......))))..(((....)))))))))).))).))).....(((....)))......................((........)).)))).....  reads  mm  sample
    ..uuuuugaagauguauugag...                    3    0  LT-3
    ..................gagcagucaaauugaauggaugau...  1    0  LT-3
    ......................Cgucaaauugaauggaug...   1    1  LCK-2
    ......................agucaaauugaauggaug...   34   0  LT-3
    ......................agucaaauugaauggauU...   3    1  LT-3
    ......................agucaaauugaauggaug...   56   0  LT-2
    ......................agucaaauugaauggaug...   68   0  LCK-2
    ......................agucCaauugaauggaug...   1    1  LT-2
    ......................agucaaauugaauggaug...   27   0  LCK-3
    ......................agucaaauugaaugNaug...   2    1  LCK-1
    ......................agucaaauugaauggauU...   1    1  LT-2
    ......................agucaaauugCauggaug...   1    1  LCK-2
    ......................agucaaCuugaauggaug...   1    1  LT-2
    ......................agucaaauugaauggaug...   91   0  LCK-1
    ......................agucaaauugaauggaug...   18   0  LT-1
    ......................agucaaauugaauggauga...  126  0  LT-3
    ......................agucaaauugaCuggauga...  1    1  LCK-2
    ......................agucaCauugaauggauga...  1    1  LT-2
    ......................Cgucaaauugaauggauga...  1    1  LT-2
    ......................agucaaauugaauggauUa...  2    1  LT-2
    ......................agucaaauugaaugNauga...  1    1  LCK-1
    ......................agucaaauugaauggauga...  41   0  LT-1
    ......................agucaaauugaauggaugC...  1    1  LT-3
    ......................agucaaauugaauggauga...  186  0  LCK-1
    ......................agucaaauCgaauggauga...  1    1  LT-1
    ......................agucCaauugaauggauga...  1    1  LCK-1
    ......................aAucaaauugaauggauga...  1    1  LCK-1
    ......................agucaaauugaauggaugU...  1    1  LT-3
    ......................agucaaauugaauggauga...  117  0  LCK-3
    ......................agucaaauugaauggauga...  147  0  LT-2
    ......................Cgucaaauugaauggauga...  1    1  LT-3
    ......................agucaaauugaauggauga...  180  0  LCK-2
    ......................agucaaauugaauggaugau... 24   0  LCK-3
```

Mature Star

```
aauuuuugaagauguauugagcagucaaauugaauggaugauugcaacaucuauucaauuugacucguuuuuuaucaucaaauuggcauacucacugaaacuuuuuggugacaaauaggaccacacaggacuagaaaucacccauuacugaagaagcaucaggacugaaaacagaagaagacagcgagacaaauggaggcccacacagaagacuuaaaacagaagagaccaauaaaaaggaccaaaaag
......................agucaaCuugaauggaugau...............................................................................................................................................................................   1     1   LT-2
......................agucaaauugaauggaugUu...............................................................................................................................................................................   4     1   LT-2
......................agucaaauugaauggaugau...............................................................................................................................................................................   6     0   LT-1
......................agucaaauugaauggaugau...............................................................................................................................................................................   14    0   LT-2
......................agucaaauugaauggaugau...............................................................................................................................................................................   14    0   LT-3
......................agNcaaauugaauggaugau...............................................................................................................................................................................   1     1   LT-2
......................agucaaauugaaugNaugauu..............................................................................................................................................................................   1     1   LCK-1
......................agucaaauugaauggaugauu..............................................................................................................................................................................   18    0   LCK-1
......................agucaaauugaauggaugCuu..............................................................................................................................................................................   1     1   LCK-2
......................agucaaauugaauggaugauu..............................................................................................................................................................................   11    0   LT-3
......................agucaaauugaauggaugauu..............................................................................................................................................................................   56    0   LCK-3
......................agucaaauugaauggaugauu..............................................................................................................................................................................   8     0   LT-1
......................agucaaauugaauggaugauu..............................................................................................................................................................................   18    0   LCK-2
......................agucaaauugaauggaugauu..............................................................................................................................................................................   11    0   LT-2
......................agucaaauugaauggaugauug.............................................................................................................................................................................   52    0   LT-3
......................agucaaauugaauggaugauug.............................................................................................................................................................................   45    0   LT-1
......................Cgucaaauugaauggaugauug.............................................................................................................................................................................   1     1   LCK-3
......................agucaaauugaauggauCauug.............................................................................................................................................................................   1     1   LT-3
......................Ggucaaauugaauggaugauug.............................................................................................................................................................................   1     1   LT-3
......................agucaaauugaauggaugauug.............................................................................................................................................................................   201   0   LCK-3
......................Cgucaaauugaauggaugauug.............................................................................................................................................................................   1     1   LCK-1
......................agucaaauugaauNgaugauug.............................................................................................................................................................................   1     1   LCK-1
......................agucaaauugaauggaugauug.............................................................................................................................................................................   95    0   LT-2
......................agucaaauugaauggaugauuU.............................................................................................................................................................................   2     1   LT-2
......................agucaaauugaauggaugauuU.............................................................................................................................................................................   2     1   LT-3
......................agucaaauugaauggaugauug.............................................................................................................................................................................   153   0   LCK-1
......................aAucaaauugaauggaugauug.............................................................................................................................................................................   1     1   LT-1
......................aAucaaauugaauggaugauug.............................................................................................................................................................................   1     1   LT-2
......................agCcaaauugaauggaugauug.............................................................................................................................................................................   1     1   LT-2
......................agucaCauugaauggaugauug.............................................................................................................................................................................   1     1   LCK-1
......................agucaaauugaCuggaugauug.............................................................................................................................................................................   1     1   LT-2
......................agucaaauugaauggaugauug.............................................................................................................................................................................   137   0   LCK-2
......................agucaaauugaaugNaugauug.............................................................................................................................................................................   2     1   LCK-1
......................agucaaauugaauggaugauugc............................................................................................................................................................................   2     0   LT-3
......................agucaaauugaauggaugauugA............................................................................................................................................................................   5     1   LT-3
......................agucaaauugaauggaugauugc............................................................................................................................................................................   8     0   LCK-3
......................agucaaauugaauggaugauugc............................................................................................................................................................................   4     0   LT-1
......................agucaaauugaauggaugauugA............................................................................................................................................................................   1     1   LT-1
......................agucaaauugaauggaugauugU............................................................................................................................................................................   1     1   LT-3
......................agucaaauugaauggaugauugc............................................................................................................................................................................   6     0   LT-2
......................agucaaauugaauggaugauugU............................................................................................................................................................................   4     1   LT-2
......................agucaaauugaauggaugauugca...........................................................................................................................................................................   3     0   LT-3
......................agucaaauugaauggaugauugca...........................................................................................................................................................................   3     0   LT-2
......................agucaaauugaauggaugauugca...........................................................................................................................................................................   3     0   LT-1
......................agucaaauugaauggaugauugcaac.........................................................................................................................................................................   7     0   LT-3
......................agucaaauugaauggaugauugcaac.........................................................................................................................................................................   8     0   LT-2
......................agucaaauugaauggaugauugcaac.........................................................................................................................................................................   4     0   LT-1
......................agucaaauugaauggaugauugcaaca........................................................................................................................................................................   80    0   LCK-1
......................agucaaauugaauggaugauugcaaca........................................................................................................................................................................   19    0   LCK-3
......................aNucaaauugaauggaugauugcaaca........................................................................................................................................................................   1     1   LCK-2
......................agucaaauugaauggaugauugcaaca........................................................................................................................................................................   76    0   LCK-2
......................agucaaauugaauggaugauugcaaca........................................................................................................................................................................   9     0   LT-1
......................agucaaauugaauggaugauugcaaca........................................................................................................................................................................   6     0   LT-3
......................agucaaauugaauggaugauugcaaca........................................................................................................................................................................   5     0   LT-2
......................agucaaauugaaugNaugauugcaaca........................................................................................................................................................................   1     1   LCK-1
......................agucaaauugaauggaugauugcaacau.......................................................................................................................................................................   1     0   LCK-3
......................agucaaauugaauggaugauugcaacUu.......................................................................................................................................................................   5     1   LCK-1
......................agucaaauugaauggaugauugcaacUu.......................................................................................................................................................................   4     1   LCK-2
......................agucaaauugaauggaugauugcaacau.......................................................................................................................................................................   3     0   LT-1
......................agucaaauugaauggaugauugcaacauc......................................................................................................................................................................   2     0   LT-1
......................agucaaauugaauggaugauugcaacauc......................................................................................................................................................................   3     0   LT-2
......................agucaaauugaauggaugauugcaacauc......................................................................................................................................................................   1     0   LCK-3
......................agucaaauugaauggaugauugcaacauU......................................................................................................................................................................   1     1   LT-2
.......................gucaaauugaauggauga................................................................................................................................................................................   4     0   LCK-3
.......................gucaaauugaauggauga................................................................................................................................................................................   1     0   LT-1
.......................gucaaauugaauggauga................................................................................................................................................................................   6     0   LT-2
.......................gucaaauugaauggauga................................................................................................................................................................................   7     0   LT-3
.......................gucaaauugaauggaugauu..............................................................................................................................................................................   5     0   LT-2
.......................gucaaauugaauggaugauug.............................................................................................................................................................................   12    0   LCK-3
.......................gucaaauugaauggaugauug.............................................................................................................................................................................   1     0   LT-1
```

Mature **Star**

aauuuuugaagauguauugagcagucaaauugaauggaugauugcaacaucuauucaauuugacucguuuuuuaucaucaaauuggcauacucacugaaacuuuuuggugacaaauaggaccacacaggacuagaaaucacccauuacugaagaagcaucaggacugaaaacagaagaagacagcgagacaaauggaggcccacacagaagacuuaaaacagaagagaccaauaaaaaggaccaaaaaag			
.......................gucaaauugaauggaugauug..	3	0	LT-2
.......................gucaaauugaauggaugauugcaaca...	12	0	LCK-1
.......................gucaaauugaauggaugauugcaaca...	3	0	LT-1
.......................gucaaauugaauggaugauugcaaca...	13	0	LCK-2
........................ucaaauugaauggaugau..	1	0	LT-2
........................ucaaauugaauggaugau..	3	0	LT-3
........................ucaaauugaauggaugauu...	5	0	LT-3
........................ucaaauugaauggaugauu...	1	0	LT-1
........................ucaaauugaauggaugauu...	5	0	LT-2
........................ucaaauugaauggaugauu...	8	0	LCK-3
........................ucaaauugaauggaugauug..	4	0	LT-3
........................ucaaauugaauggaugauug..	3	0	LT-2
........................ucaaauugaauggaugauug..	1	0	LT-1
........................ucaaauugaauggaugauugU...	3	1	LT-2
........................ucaaauugaauggaugauugcaac..	1	0	LT-1
........................ucaaauugaauggaugauugcaacU...	1	1	LT-3
.........................caaauugaauggaugauu...	5	0	LT-2
.........................caaauugaauggaugauu...	1	0	LT-1
.........................caaauugCauggaugauu...	1	1	LT-2
.........................caaauugaauggaugauu...	2	0	LT-3
.........................caaauugaauggaugauugcaaca...	15	0	LCK-1
.........................caaauugaauggaugauugcaaca...	15	0	LCK-2
..........................aaauugaauggaugauugcaaca...	2	0	LT-2
...........................aauugaauggaugauugcaac..	14	0	LCK-2
...........................aauugaauggaugauugcaac..	24	0	LCK-1
...........................aauugaauggaugauugcaaca...	7	0	LCK-2
...........................aauugaauggaugauugcaaca...	1	0	LT-3
...........................aauugaauggaugauugcaaca...	10	0	LCK-1
...........................aauugaauggaugauugcaCca...	1	1	LCK-1
............................auugaauggaugauugcaac..	11	0	LCK-2
............................auugaauggaugauugcaac..	10	0	LCK-1
............................auugaauggaugauugcaaca...	1	0	LT-2
............................auugaauggaugauugcaaca...	2	0	LCK-3
.............................uugaauggaugauugcaac..	3	0	LT-2
.............................uugaauggaugauugcaaca...	1	0	LT-3
.............................uugCauggaugauugcaaca...	1	1	LCK-1
.............................uugaauggaugauugcaaca...	4	0	LCK-3
.............................uugaauggaugauuNcaaca...	1	1	LCK-1
.............................uugaauggaugauugcaaca...	26	0	LCK-1
.............................uugaauggaugauugcaaca...	25	0	LCK-2
..............................ugaauggaugauugcaac..	4	0	LCK-1
..............................ugaauggaugauugcaac..	1	0	LCK-2
..............................ugaauggaugauugcaaca...	1	0	LT-1
..............................ugaauggaugauugcaacau..	1	0	LT-3
...............................gaauggaugauugcaaca...	1	0	LT-2
................................aauggaugauugcaacauc...	1	0	LT-2
................................aauggaugauugcaacauUua...	2	1	LT-3
.................................auggaugauugcaacauU...	9	1	LCK-3
.................................auggaugauugcaacauc...	1	0	LT-1
.................................auggaugauugcaacauc...	3	0	LT-3
.................................auggaugauugcaacauUu..	1	1	LT-3
.................................auggaugauugcaacauUuauuc..	1	1	LT-3
.................................auggaugauugcaacauUuauuc..	2	1	LT-1
.................................auggaugauugcaacauUuauuc..	6	1	LCK-3
..................................uggaugauugcaacauUua...	1	1	LT-3
..................................uggaugauugcaacaucuauuc..	1	0	LT-1
.....................................augauugcaacauUuauu...	1	1	LT-3
....gauugcaacaucuauucaauuugacucguu...	1	0	LT-3
....uugcaacaucuauucaauuu...	2	0	LT-3
....uugcaacaucuauucaauuugacu...	3	0	LT-2
....uugcaacaucuauucaauuugacu...	1	0	LT-3
....uugcaacauUuauucaauuugacu...	3	1	LT-1
....uugcaacauUuauucaauuugacuc..	1	1	LT-3
....uugcaacaucuauucaauuugacuc..	1	0	LT-1
....ugcaacaucuauucaauuugacu...	1	0	LT-3
....Ucaacaucuauucaauuugacu...	1	1	LCK-3
....gcaacaucuauucaauuugacucA...	2	1	LT-1
....gcaacaucuauucaauuugacucguu...	1	0	LT-1
....caacaucuauucaauuugaU..	3	1	LT-2
....caacaucuauucaauuugac..	3	0	LT-3

Mature Star

aauuuuugaagauguauugagcagucaaauugaauggaugauugcaacaucuauucaauuugacucguuuuuuaucaucaaauuggcauacucacugaaacuuuuuggugacaaauaggaccacacaggacuagaaaucacccauuacugaagaagcaucaggacugaaaacagaagaagacagcgagacaaauggaggcccacacagaagacuuaaaacagaagagaccaauaaaaaggaccaaaaaag

caacaucuauucaauuugac	2	0	LT-1
caacaucuauucaauuugac	4	0	LT-2
caacaucCauucaauuugacu	1	1	LT-2
caacaucuauucaauuugaAu	1	1	LCK-1
caacaucuauucaauuugacu	93	0	LT-3
caacaucuCuucaauuugacu	1	1	LT-3
caacaucuauucGauuugacu	1	1	LT-3
caacauUuauucaauuugacu	5	1	LT-2
caCcaucuauucaauuugacu	1	1	LCK-1
caacaucuauucaauuugacu	114	0	LCK-2
caacaucuauucaaNuugacu	1	1	LCK-1
caacaucuauucaNuuugacu	1	1	LCK-1
caCcaucuauucaauuugacu	1	1	LT-3
caacaucuCuucaauuugacu	1	1	LT-2
caacaucuauucaauuugacu	115	0	LT-2
caCcaucuauucaauuugacu	1	1	LT-2
cCacaucuauucaauuugacu	1	1	LT-2
caacaucuauucaauuugacu	50	0	LT-1
caacaucuauucaCuuugacu	1	1	LCK-3
caacauUuauucaauuugacu	3	1	LT-3
caacCucuauucaauuugacu	1	1	LT-2
caacaucuauucaauuugacu	138	0	LCK-3
caacaucuauucaauuugacu	76	0	LCK-1
caacaucuauucaaGuugacu	1	1	LCK-1
caacaucuauucaGuuugacu	1	1	LCK-1
caacCucuauucaauuugacuc	1	1	LT-2
caacaucuauucaauuugacuc	52	0	LCK-3
caacaucuauucaauuugacuc	30	0	LCK-1
caCcaucuauucaauuugacuc	1	1	LT-3
caacauUuauucaauuugacuc	2	1	LT-2
caacaucuauucaauuugacuc	24	0	LT-3
caacaucuauucaauuugacuc	30	0	LT-1
caacauUuauucaauuugacuc	6	1	LT-3
caacauUuauucaauuugacuc	8	1	LCK-3
caacauUuauucaauuugacuc	3	1	LT-1
caacaucuauucaauuugacuc	41	0	LT-2
caacaucuaCucaauuugacuc	1	1	LT-2
caacaucuauucaauuugacuU	1	1	LT-1
caacaucuauucaauuugacuc	30	0	LCK-2
caacaucuCuucaauuugacuc	1	1	LCK-3
caacaucuauucaauuugacucg	3	0	LT-1
caacaucuauucaauuugacucg	1	0	LT-2
caacauUuauucaauuugacucg	3	1	LT-1
caacaucuauucaauuugacucg	1	0	LCK-3
caacaucuauucaauuugacucU	5	1	LT-3
caacaucuauucaauuugacucC	2	1	LT-1
caacaucuauucaauuugacucg	5	0	LT-3
caacaucuauucaauuugacucU	2	1	LT-2
caacaucuauucaauuugacucU	3	1	LT-1
caacauUuauucaauuugacucg	1	1	LT-3
caacaucuauucaauuugacucgu	2	0	LT-1
caacaucuauucaauuugacucgu	2	0	LT-3
aacaucuauucaauuugaU	1	1	LT-3
aacaucuauucaauuugaUu	1	1	LT-1
aacaucuauucCauuugacu	1	1	LCK-1
Cacaucuauucaauuugacu	1	1	LCK-1
aacaucuauucaauuugacu	4	0	LT-2
aCcaucuauucaauuugacu	1	1	LT-2
aacaucuauucaauuugacu	8	0	LT-3
aacaucuauucaauuugacu	14	0	LCK-3
aacaucuauucaauuGgacu	1	1	LT-2
aacaucuauucaauuugacA	1	1	LT-2
aacaucuauucaauuugacu	16	0	LCK-1
aacauUuauucaauuugacu	1	1	LT-3
aacaucuauucaauuugacu	19	0	LCK-2
aacaucuauucaauuugacu	1	0	LT-1
aacaucuauucaauuugacuc	25	0	LCK-1
aacaucuauucaauuugacuc	11	0	LT-2
aacaucuauucaauuugacuc	29	0	LCK-2
aacaucuauucaauuugacuc	3	0	LT-1

```
                      Mature                Star
aauuuuugaagauguauugagcagucaaauugaauggaugauugcaacaucuauucaauuugacucguuuuuuaucaucaaauuggcauacucacugaaacuuuuuggugacaaauaggaccacacaggacuagaaaucacccauuacugaagaagcaucaggacugaaaacagaagaagacagcgagacaaauggaggcccacacagaagacuuaaaacagaagagaccaauaaaaaggaccaaaaaag
.............................................aacaucuauucaauuugacuc........................................................................................................................................................................................    5    0    LT-3
.............................................aacaucuauucaauuugacuc........................................................................................................................................................................................    2    0    LCK-3
.............................................aacaucuauucaaNuugacuc........................................................................................................................................................................................    1    1    LCK-1
.............................................aacaucuauucaauuugacucU.......................................................................................................................................................................................    1    1    LT-1
.............................................aacaucuauucaauuugacucg.......................................................................................................................................................................................    4    0    LT-1
.............................................aacaucuauucaauuugacucU.......................................................................................................................................................................................    5    1    LCK-3
.............................................aacaucuauucaauuugacucg.......................................................................................................................................................................................    4    0    LT-2
.............................................aacaucuauucaauuugacucg.......................................................................................................................................................................................    7    0    LT-3
.............................................aacauUuauucaauuugacucg.......................................................................................................................................................................................    1    1    LT-3
.............................................aacauUuauucaauuugacucg.......................................................................................................................................................................................    1    1    LT-1
.............................................aacaucuauucaauuugacucC.......................................................................................................................................................................................    1    1    LT-3
.............................................aacaucuauucaauuugacucgA......................................................................................................................................................................................    1    1    LT-3
..............................................acauUuauucaauuugac..........................................................................................................................................................................................    1    1    LT-1
..............................................acauUuauucaauuugacu.........................................................................................................................................................................................    1    1    LT-2
..............................................acaucuauucaauuugacu.........................................................................................................................................................................................    14   0    LCK-3
..............................................acaucuauucaauuugacu.........................................................................................................................................................................................    28   0    LT-2
..............................................acaucuauucaauuugacu.........................................................................................................................................................................................    11   0    LT-1
..............................................acauUuauucaauuugacu.........................................................................................................................................................................................    4    1    LT-3
..............................................acaucuauucaauuugacu.........................................................................................................................................................................................    20   0    LT-3
..............................................acaucuauucaauuugacuc........................................................................................................................................................................................    20   0    LT-3
..............................................acCucuauucaauuugacuc........................................................................................................................................................................................    1    1    LT-1
..............................................acaucuauucaauuugacuU........................................................................................................................................................................................    2    1    LT-1
..............................................acaucuauucaauuugacuU........................................................................................................................................................................................    3    1    LT-2
..............................................acaucuauucaauuugacuc........................................................................................................................................................................................    16   0    LCK-3
..............................................acaucuauucaauuugacuc........................................................................................................................................................................................    12   0    LT-1
..............................................acauUuauucaauuugacuc........................................................................................................................................................................................    3    1    LT-1
..............................................acauUuauucaauuugacuc........................................................................................................................................................................................    1    1    LT-2
..............................................acaucuauucaauuugacuU........................................................................................................................................................................................    12   1    LCK-3
..............................................acauUuauucaauuugacuc........................................................................................................................................................................................    3    1    LT-3
..............................................acaucuauucaauuugacuc........................................................................................................................................................................................    10   0    LT-2
..............................................acauUuauucaauuugacucg.......................................................................................................................................................................................    5    1    LT-3
..............................................acauUuauucaauuugacucg.......................................................................................................................................................................................    5    1    LT-2
..............................................acaucuauucaauuugacucg.......................................................................................................................................................................................    11   0    LT-2
..............................................acauUuauucaauuugacucg.......................................................................................................................................................................................    5    1    LT-1
..............................................acaucuauucaauuugacucU.......................................................................................................................................................................................    3    1    LT-3
..............................................acaucuauucaauuugacucg.......................................................................................................................................................................................    8    0    LCK-3
..............................................acaucuauucaauuugacucU.......................................................................................................................................................................................    2    1    LT-1
..............................................acaucuauucaauuugacucg.......................................................................................................................................................................................    17   0    LT-1
..............................................acaucuauucaauuugacucU.......................................................................................................................................................................................    5    1    LT-2
..............................................acaucuauucaauuugacucg.......................................................................................................................................................................................    27   0    LT-3
..............................................acauUuauucaauuugacucg.......................................................................................................................................................................................    15   1    LCK-3
..............................................acaucuauucaauuugacucgA......................................................................................................................................................................................    2    1    LT-3
..............................................acaucuauucaauuugacucgC......................................................................................................................................................................................    1    1    LT-1
..............................................acaucuauucaauuugacucgC......................................................................................................................................................................................    1    1    LT-3
..............................................acaucuauucaauuugacucUuu.....................................................................................................................................................................................    1    1    LT-3
..............................................acaucuauucaauuugacucgCu.....................................................................................................................................................................................    2    1    LT-2
..............................................acauUuauucaauuugacucguu.....................................................................................................................................................................................    1    1    LT-1
..............................................acaucuauucaCuuugacucguu.....................................................................................................................................................................................    1    1    LT-1
..............................................acaucuauucaauuugacucgCu.....................................................................................................................................................................................    1    1    LT-1
..............................................acaucuauucaauuugacucguA.....................................................................................................................................................................................    1    1    LT-3
..............................................acaucuauucaauuugacucguu.....................................................................................................................................................................................    1    0    LT-1
...............................................cauUuauucaauuugacu.........................................................................................................................................................................................    5    1    LT-2
...............................................caucuauucaauuugacu.........................................................................................................................................................................................    50   0    LCK-3
...............................................cauUuauucaauuugacu.........................................................................................................................................................................................    12   1    LCK-1
...............................................caucuauucaauuugacu.........................................................................................................................................................................................    42   0    LT-3
...............................................cauUuauucaauuugacu.........................................................................................................................................................................................    5    1    LCK-2
...............................................caucuauucaauuugacu.........................................................................................................................................................................................    50   0    LT-2
...............................................caucuauucaauuugacu.........................................................................................................................................................................................    37   0    LCK-1
...............................................caucuauucaauuugaAu.........................................................................................................................................................................................    1    1    LCK-3
...............................................cauUuauucaauuugacu.........................................................................................................................................................................................    3    1    LCK-3
...............................................caucuauucaauuugacu.........................................................................................................................................................................................    15   0    LT-1
...............................................cauUuauucaauuugacu.........................................................................................................................................................................................    1    1    LT-1
...............................................caucuauucaauuugacu.........................................................................................................................................................................................    47   0    LCK-2
...............................................cauUuauucaauuugacu.........................................................................................................................................................................................    1    1    LT-3
...............................................cCucuauucaauuugacu.........................................................................................................................................................................................    1    1    LT-2
...............................................cauUuauucaauuugacuc........................................................................................................................................................................................    3    1    LT-1
...............................................caucuauucaauuugacuc........................................................................................................................................................................................    10   0    LT-1
...............................................cCucuauucaauuugacuc........................................................................................................................................................................................    1    1    LT-2
...............................................cauUuauucaauuugacuc........................................................................................................................................................................................    6    1    LT-3
...............................................cauUuauucaauuugacuc........................................................................................................................................................................................    9    1    LT-2
```

Mature Star

aauuuuugaagauguauugagcagucaaauugaauggaugauugcaacaucuauucaauuugacucguuuuuuaucaucaaauuggcauacucacugaaacuuuuuggugacaaauaggaccacacaggacuagaaaucacccauuacugaagaagcaucaggacugaaaacagaagaagacagcgagacaaauggaggcccacacagaagacuuaaaacagaagagaccaauaaaaaggaccaaaaaag

cCucuauucaauuugacuc	1	1	LCK-3
caucuauucaauuugacuc	44	0	LT-2
caucuauucaauuugacuc	18	0	LT-3
cauUuauucaauuugacuc	14	1	LCK-3
caucuauucaauuugacuc	17	0	LCK-3
caucuCuucaauuugacuc	1	1	LT-3
cauUuauucaauuugacucg	3	1	LT-3
caucuauucaauuugacucU	1	1	LT-2
caucuauucaauuugacucg	13	0	LCK-3
caucuauucaauuugacucU	2	1	LT-1
cauUuauucaauuugacucg	9	1	LT-2
caucuauucaauuugacucg	2	0	LT-1
caucuauucaauuugacucg	3	0	LT-3
caucuauucaauuugacucC	2	1	LT-3
caucuauucaauuugacucU	2	1	LCK-3
caucuauucaauuugacucU	4	1	LT-3
cauUuauucaauuugacucgu	2	1	LT-1
caucuauucaauuugacucgu	2	0	LT-1
cauUuauucaauuugacucgu	2	1	LCK-3
caucuauucaauuugacucgu	4	0	LT-3
caucuauucaauuugacucgA	2	1	LT-3
caucuauucaauuugacucgu	3	0	LT-2
cauUuauucaauuugacucgu	2	1	LT-3
cauUuauucaauuugacucguu	2	1	LT-3
caucuauucaauuugacucguu	10	0	LT-3
caucuauucaauuugacucUuu	3	1	LT-3
caucuauucaauuugacucguu	18	0	LT-2
caucuauucaauuugacucUuu	7	1	LT-2
caucuauucaauuugacucguu	11	0	LCK-3
caucuauucaauuugacucguC	1	1	LT-3
cauUuauucaauuugacucguu	6	1	LT-2
cauUuauucaauuugacucguu	2	1	LT-1
caucuauucaauuugacucAuu	1	1	LT-3
caucuauucaauuugacucguu	6	0	LT-1
caucuauucaauuugacucguuC	3	1	LT-2
caucuauucaauuugacucguuu	2	0	LT-1
caucuauucaauuugacucguuu	2	0	LT-3
caucuauucaauuugacucguuu	3	0	LT-2
caucuauucaauuugacucguuA	1	1	LT-1
auUuauucaauuugacuc	1	1	LT-1
aucuauucaauuugacuc	1	0	LT-2
aucuauucaauuugacuc	1	0	LT-3
aucuauucaauuugacucguA	5	1	LCK-3
ucuauucaauuugacucg	1	0	LT-3
ucuauucaauuugacucC	1	1	LT-2
cuauucaauuugacucUuu	8	1	LCK-3
cuauucaauuugacucUuu	1	1	LT-2
cuauucaauuugacucguuuuu	1	0	LT-2
ucaaauuggcauacucacugaaacuuuuu	1	0	LT-2
aauuggcauacucacugaaacuuuu	3	0	LT-2
aauuggcauacucacugaaacuuuuu	1	0	LT-3
aauuggcauacucacugaaacuuuuu	1	0	LT-1
auuggcauacucacugaaaUuuuuu	1	1	LT-3
auuggcauacucacugaaacuuuuA	1	1	LT-3
uuggcauacucacugaaacuu	1	0	LT-3
uuggcauacucacugaaacuuu	1	0	LT-1
uggcauacucacugaaacuuuu	1	0	LT-3
gcauacucacugaaacuuuu	1	0	LT-3
cauacucacugaaacuuuu	1	0	LT-3
ugacaaauaggaccacaca	1	0	JCK-3
cacacaggacuagaaaucacc	1	0	JCK-2
cCgaagacuuaaaacagaagag	1	1	LT-1
agacuuaaaacagaaCag	1	1	LT-3
